# Karyotype Evolution in Triatominae (Hemiptera, Reduviidae): The Role of Chromosomal Rearrangements in the Diversification of Chagas Disease Vectors

**DOI:** 10.3390/ijms24076350

**Published:** 2023-03-28

**Authors:** Yago Visinho dos Reis, Jader de Oliveira, Fernanda Fernandez Madeira, Amanda Ravazi, Ana Beatriz Bortolozo de Oliveira, Isadora da Silva Bittinelli, Luiza Maria Grzyb Delgado, Maria Tercília Vilela de Azeredo-Oliveira, João Aristeu da Rosa, Cleber Galvão, Kaio Cesar Chaboli Alevi

**Affiliations:** 1 Instituto de Biociências de Botucatu, Universidade Estadual Paulista “Júlio de Mesquita Filho” (UNESP), Rua Dr. Antônio Celso Wagner Zanin, 250, Distrito de Rubião Junior, Botucatu 18618-689, SP, Brazil; 2 Laboratório de Entomologia em Saúde Pública, Faculdade de Saúde Pública, Universidade de São Paulo (USP), Av. Dr. Arnaldo 715, São Paulo 01246-904, SP, Brazil; 3 Laboratório de Biologia Celular, Instituto de Biociências, Universidade Estadual Paulista “Júlio de Mesquita Filho” (UNESP), Letras e Ciências Exatas, Rua Cristóvão Colombo 2265, São José do Rio Preto 15054-000, SP, Brazil; 4 Laboratório de Parasitologia, Faculdade de Ciências Farmacêuticas, Universidade Estadual Paulista “Júlio de Mesquita Filho” (UNESP), Rodovia Araraquara-Jaú km 1, Araraquara 14801-902, SP, Brazil; 5 Laboratório Nacional e Internacional de Referência em Taxonomia de Triatomíneos, Instituto Oswaldo Cruz (FIOCRUZ), Av. Brasil 4365, Pavilhão Rocha Lima, sala 505, Rio de Janeiro 21040-360, RJ, Brazil

**Keywords:** ChromoSSE, experimental crosses, phylogenetic analysis, karyotypic isolation

## Abstract

Several cytogenetic studies have already been performed in Triatominae, such that different karyotypes could be characterized (ranging from 2n = 21 to 25 chromosomes), being the changes in the number of chromosomes related mainly to fusion and fission events. These changes have been associated with reproductive isolation and speciation events in other insect groups. Thus, we evaluated whether different karyotypes could act in the reproductive isolation of triatomines and we analyzed how the events of karyotypic evolution occurred along the diversification of these vectors. For this, experimental crosses were carried out between triatomine species with different karyotypes. Furthermore, based on a phylogeny with 88 triatomine taxa (developed with different molecular markers), a reconstruction of ancestral karyotypes and of anagenetic and cladogenetic events related to karyotypic alterations was performed through the ChromoSSE chromosomal evolution model. All crosses performed did not result in hybrids (prezygotic isolation in both directions). Our modeling results suggest that during Triatominae diversification, at least nine cladogenetic events may be associated with karyotype change. Thus, we emphasize that these alterations in the number of chromosomes can act as a prezygotic barrier in Triatominae (karyotypic isolation), being important evolutionary events during the diversification of the species of Chagas disease vectors.

## 1. Introduction

Chagas disease, caused by the protozoan *Trypanosoma cruzi* (Chagas, 1909) (Kinetoplastida, Trypanosomatidae), has no cure in the chronic phase and affects about seven million people worldwide [1,2]. The main form of transmission of this protozoan is vectorial, through the feces/urine of infected triatomines (Hemiptera, Triatominae) [3,4], since these insects are obligatory hematophagous and have the habit of defecating/urinating during the blood meal [3,4]. Currently, 157 species of triatomines are known (three are fossils), which are distributed in 18 genera and five tribes [5,6,7].

The generation of hybrids is relatively common between the species of this subfamily, with the main evolutionary events responsible for the decline of the hybrid lineage (postzygotic barriers) being inviability (offspring mortality before reaching adulthood) [8], sterility (partially or completely infertile offspring) [9,10] and the collapse of the hybrid (high offspring mortality from the second generation) [11,12]. Several studies involving experimental crosses have already been carried out among the triatomines, focusing mainly on taxonomic [8,11,13,14,15,16,17,18,19,20,21,22,23] and epidemiological aspects [24,25,26,27]. On the other hand, although prezygotic barriers are less frequent and, generally, are present in only one direction of the crossings, as observed between *Rhodnius colombiensis* Mejia, Galvão and Jurberg, 1999 and *R. pallescens* Barber, 1932 [28], *Triatoma pseudomaculata* Corrêa and Espínola, 1964 and *T. infestans* (Klug, 1834) [29], *T. delpontei* Romaña and Abalos, 1947 and *T. platensis* Neiva, 1913 [30] and *T. longipennis* Usinger, 1939 and *T. mopan* Dorn et al., 2018 [17], they may be present between species that are very distant from the phylogenetic point of view [29,31] and between species of different genera (*Triatoma* Laporte, 1832 × *Panstrongylus* Berg, 1879, *Triatoma* × *Rhodnius* Stål, 1859 and *Rhodnius* × *Psammolestes* Bergroth, 1911 [18,23,31]).

Recently, experimental crosses between triatomine species with different chromosome numbers were performed, and it was demonstrated that there is prezygotic isolation [18,32]. Based on these observations, the authors suggested karyotypic variation may be an important factor in the reproductive isolation of these insects [18,32]. In Lepidoptera, for example, it has been suggested that in some genera, karyotypic changes may be related to speciation events [33]. Thus, it is possible that these numerical changes also played a relevant role in the diversification of Triatominae species.

Ueshima [34], based on the modal number, proposed that the ancestral karyotype of triatomines would be 2n = 22 chromosomes (20A + XY, in males; 20A + XX, in females), so that the karyotypic variation present in the current species (2n = 21 to 25 chromosomes) would have arisen after chromosomal fission and fusion events (although aneuploidy events related to chromosomal loss cannot be ruled out) [34,35,36,37,38,39]. Several studies involving the evolution of the karyotype in Triatominae were carried out from inferences made based on the phylogenetic relationships of the species [36,37,38]. However, there are specific models for chromosomal evolution studies, such as ChromEvol [40,41] and ChromoSSE [42], which can help to understand how these changes occurred throughout the evolutionary process of these vectors.

In general, the application of the ChromEvol model allows the changes in the number of chromosomes along the branches of a phylogenetic tree (anagenesis) to be evaluated, also allowing to infer the ancestral karyotype at each phylogeny node [41]. The ChromoSSE model allows the evaluation, under a Bayesian approach, not only of anagenetic processes, but also of cladogenetic events related to karyotypic changes [42].

Based on the above, we performed several interspecific crosses to evaluate the role of the karyotype in the reproductive isolation of triatomine species. In addition, we evaluated the implications of possible anagenesis and cladogenesis events related to changes in the number of chromosomes throughout the evolutionary process of Chagas disease vectors.

## 2. Results and Discussion

**Figure 1:**
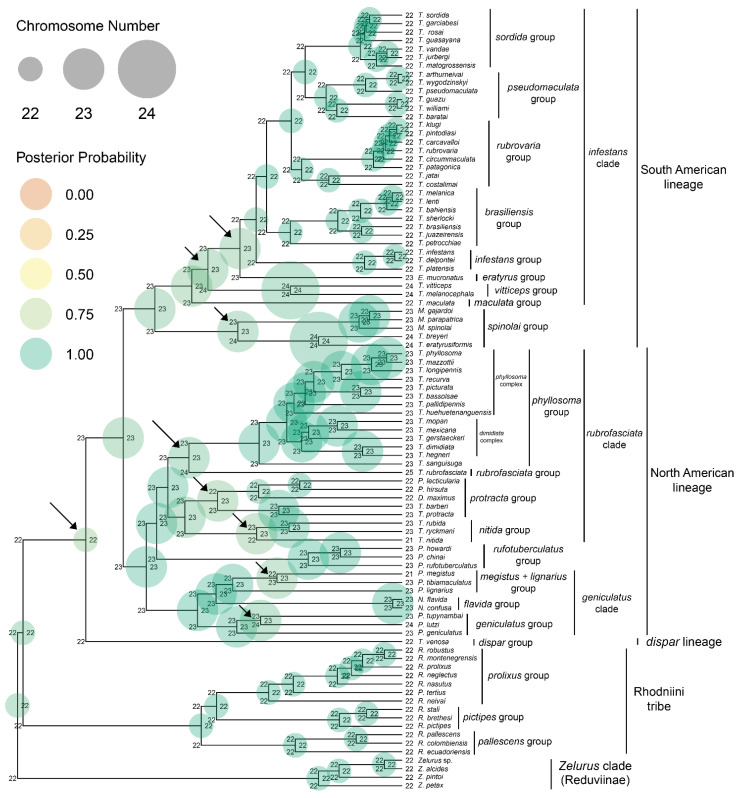
Maximum a posteriori (MAP) estimate of the chromosome number in triatomine ancestors (nodes). The number observed on the “shoulders” shows the karyotype of each lineage just after cladogenesis. Arrows indicate where karyotype alterations may have played a role in cladogenesis.

All interspecific crosses performed between species with different chromosome numbers did not result in hybrids (Table 1). On the other hand, intraspecific crosses (control) showed hatching rates ranging from 51 to 86% (Table 1). Furthermore, the Bayesian phylogeny obtained (Figure S1) and used in studies related to karyotypic evolution (Figure 1) showed a topology similar to that of the main phylogenetic reconstructions for Triatominae available in the literature [43,44], being most clusters with good support (posterior probability > 0.8).

In general, the subfamily Triatominae and the Rhodniini and Triatomini tribes were recovered as monophyletic groups (Figure 1). In addition, all species groups were recovered as monophyletic, satisfying the proposal by Justi et al. [43] who consider that the clusters (groups, complexes and subcomplexes) should represent natural groups. Curiously, *T. guasayana* Wygodzinsky and Abalos, 1949 was recovered together with the *sordida* group and not with the *rubrovaria* group (Figure 1) (different from what was recently proposed by Belintani et al. [45]) and the *rufotuberculatus* group did not group with the rest of the genus *Panstrongylus* (Figure 1) (different from what was recently observed by Bittinelli et al. [46]). In addition, our results demonstrated that the species of the *spinolai* complex are more closely related to triatomines from South America (Figure 1) (as noted by Justi et al. [43] and Pita et al. [47]).

As previously mentioned, the number of chromosomes in the Triatominae subfamily ranges from 21 to 25 (in males): 2n = 21 (18A + X_1_X_2_Y), 2n = 22 (20A + XY), 2n = 23 (20A + X_1_X_2_Y), 2n = 24 (20A + X_1_X_2_X_3_Y) and 2n = 25 (22A + X_1_X_2_Y) [37,38,39]. The phylogenetic reconstruction recovered the ancestral karyotype of triatomines as 2n = 22 (PP = 1.0) (Figure 1), corroborating the proposal made by Ueshima [34] based on the modal number. However, the analysis also suggested that the current species of the Triatomini tribe with 22 chromosomes (with the exception of the *dispar* group) may have arisen from ancestors who already had X chromosome fragmentation (Figure 1). In addition, nine cladogenetic events related to changes in chromosome number may have occurred in Triatominae (Figure 1, arrows).

Chromosomal changes associated with cladogenetic events occurred in both autosomes and sex chromosomes. It is important to emphasize that, in species in which there was an increase or a decrease in a pair of autosomes [*T. nitida* Usinger, 1939, *T. rubrofasciata* (De Geer, 1773) and *P. megistus* (Burmeister, 1835)], the model considered this change as two independent events for each homologous chromosome: for *T. nitida* and *P. megistus*, from 23 to 22 (as a cladogenetic event) and later from 22 to 21 (anagenetic); for *T. rubrofasciata*, from 23 to 24 (cladogenetic) and from 24 to 25 (anagenetic) (this was because the model considers only one modification at a time). However, as the chromosomes are organized in pairs in 2n (bivalent) cells, we considered this as just a cladogenetic event.

Our results indicated that the Rhodniini tribe is the most basal group and presents the same karyotype as the ancestor 2n = 22 (Figure 1). However, we emphasize that if species from the Cavernicolini, Bolboderini and Alberprosini tribes (which have few sequences deposited and/or have never been studied cytogenetically) were included, a different topology could have been recovered. Thus, we will discuss the cladogenetic events related to the karyotypic alterations of the Triatomini tribe (Figure 1, arrows) for the three groupings: the dispar lineage, the South American lineage and the North American lineage.

### 2.1. Dispar Lineage

The vicariance event related to the separation of the *dispar* group [represented by *T. venosa* (Stål, 1872)] from the other species of the Triatomini tribe is related to the uplift of the Western Cordillera of North America [44]. Our results suggest that the first cladogenetic event related to the fission of the X sex chromosome may have occurred during this separation (Figure 1). However, as *Belminus* Stål, 1859 species (Bolboderini tribe) were recently recovered as a sister group to Triatomini [48], we cannot rule out the possibility that the ancestor of the Triatomini tribe had X chromosome fragmentation, since species of the genus *Belminus* have 2n = 23 [36,39]. We highlight that *Belminus* spp. were not included due to the low availability of related sequences in GenBank. Thus, when considering the Bolboderini tribe (and, consequently, the ancestral karyotype 2n = 23), it is possible that the diversification of the ancestors of the *dispar* group in relation to the other species of the Triatomini tribe may be due to a fusion event (or loss) of one X chromosome.

### 2.2. North American Lineage

North American triatomines are divided into two clades and eight groups (the *phyllosoma* group is composed of the *phyllosoma* and *dimidiata* complexes) (Figure 1). Alevi et al. [38] suggested that the karyotype differences observed between the species of the *geniculatus* clade may be due to the fusion (or loss) of a pair of autosomes (in the ancestors of *P. megistus*) and the fission of the X chromosome [in the ancestors of *P. lutzi* (Neiva and Pinto, 1923)]. Our modeling results suggest that these chromosomal changes may have played a role in cladogenesis between *P. lutzi* and *P. tupynambai* Lent, 1942 (Figure 1, arrow) and between *P. megistus* and *P. tibiamaculatus* (Pinto, 1926) (Figure 1, arrow). The separation of the ancestors of *P. megistus* and *P. tibiamaculatus* has already been related to the formation of a dry corridor between the Atlantic Forest and the Amazon rainforest after the uplift of the Andes, which acted as a vicariant event [44]. Considering that allopatric species may not develop mechanisms that make hybridization between them unfeasible [49], the prezygotic isolation observed in experimental crosses between *P. megistus* and *P. tibiamaculatus* [also observed between *P. megistus* and *P. lignarius* (Walker, 1873)] (Table 1) may be related to the difference in the number of chromosomes [32]. Thus, the alteration of the karyotype in the ancestors of *P. megistus* may have contributed to the isolation between these species.

The position of the *rufotuberculatus* group in the phylogeny is quite intriguing, since it is placed outside the *geniculatus* clade and is closer to the *rubrofasciata* clade (composed of species of the genus *Triatoma*) (Figure 1). The position of *P. rufotuberculatus* (Champion, 1899) has previously been questioned [48,50,51], a greater phylogenetic proximity of this species to the *rubrofasciata* clade being observed [48,50]. Thus, further studies should be performed in order to elucidate these evolutionary relationships.

*Panstrongylus noireaui* Gil-Santana et al., 2022 was recently described from specimens initially classified as *P. rufotuberculatus* from Bolivia [7]. Phylogenetic studies have recovered *P. noireaui* as a sister species of *P. rufotuberculatus* [52]. However, in contrast to *P. rufotuberculatus* and all other *Panstrongylus* species, this species has 22 chromosomes [52]. The authors suggest that the size of the X chromosome of this species is equivalent to that of the sum of the X chromosomes (X_1_ and X_2_) of *P. rufotuberculatus*, which indicates the possible occurrence of chromosomal fusion [52]. Thus, despite *P. noireaui* was not included in our analysis, we emphasize the possibility that this chromosomal fusion had a cladogenetic role between these two species. Although the authors suggested that hybridization may occur between them [52], we believe that the difference in the number of chromosomes acts as a prezygotic barrier between *P. noireaui* and *P. rufotuberculatus*.

Cladogenetic events resulting from karyotypic changes may also have occurred in the *nitida* and *protracta* groups (Figure 1). The origin of the *T. nitida* karyotype is related to the fusion (or loss) of a pair of autosomes (as in *P. megistus*) [38]. Thus, it is possible that this change in the number of chromosomes have promoted a reproductive isolation between the ancestor of *T. nitida* and the ancestor of the lineage *T. rubida* and *T. ryckmani* (Figure 1). In the *protracta* group, the fusion (or loss) of an X chromosome in the ancestors of the *Paratriatoma* Barber, 1938 and *Dipetalogaster* Usinger, 1939 clades may have occurred in a cladogenetic manner (Figure 1), resulting in the isolation of the ancestors of these species from the ancestors that gave rise to *T. protracta* (Uhler, 1894) and *T. barberi* Usinger, 1939. Experimental crosses have already been carried out between *T. protracta* and *T. barberi*, revealing reproductive compatibility, obtaining hybrids up to the second generation [53]. Already in crosses between *T. barberi* and *T. rubida* (Uhler, 1894), hybrids were produced, but they were not viable [53]. However, in the crosses carried out between *T. protracta* and *P. lecticularia* (Stål, 1859) (previously included in the genus *Triatoma* [54]) we found the presence of a prezygotic barrier (Table 1). This reinforces the importance of karyotypic alterations in the reproductive isolation of these vectors, since even between species of these groups that are phylogenetically more distant but that present the same karyotype (*T. barberi* and *T. rubida*), there are no prezygotic barriers [53].

Regarding the *rubrofasciata* clade, the gain of a pair of autosomes in *T. rubrofasciata* [38] may also have acted as a cladogenetic event, resulting in the isolation of the ancestors of this species (Figure 1). However, several phylogenetic analyses have grouped *T. rubrofasciata* with species of the genus *Linshcosteus* Distant, 1904 and other species of Old World *Triatoma* [43,44,48], for which there are no cytogenetic data (thus, they were not included in phylogenetic studies). Justi et al. [44] suggested a Neotropical origin for the clade involving these species (25–10 Ma), with later separation of the ancestors that would originate the Old World species (from those that would give rise to *T. rubrofasciata*). Therefore, the dispersion of *T. rubrofasciata* to the locations where it is found today (more than 40 countries [55]) would have occurred only recently by ships [44,51]. On the other hand, Kieran et al. [48] observed that *T. rubrofasciata* clusters with *T. bouvieri* Larrousse, 1924 and *T. migrans* Breddin, 1903 (present in the Old World), suggesting that the origin of this species is in the Old World. Considering the possibility of hybridization after secondary contact between allopatric species [56], if Old World species have the same number of chromosomes as *T. rubrofasciata*, it is possible that natural crosses followed by introgression events may have occurred between these species, as observed in *Rhodnius* [57]. Thus, the grouping of these species observed by Kieran et al. [48] may be related to these events, and the origin of *T. rubrofasciata* is in fact neotropical, emphasizing the need for cytogenetic studies, as well as tests of experimental crosses between these species to elucidate the origin and diversification of these triatomines.

### 2.3. South American Lineage

The South American triatomines are divided into nine groups (Figure 1). The first cladogenetic event related to the variation in the number of chromosomes that may have occurred in this lineage may be associated with the diversification of the species of the *spinolai* group (Figure 1). This group is formed by *T. breyeri* Del Ponte, 1929, *T. eratyrusiformis* Del Ponte 1929 and by *Mepraia* spp. [51]. The phylogenetic position of this group within the Triatomini tribe is still controversial, as in some phylogenetic reconstructions, they are grouped in the South American lineage [as well as in our analysis (Figure 1)] [43], while in other studies, in the North American one [44,48]. Based on karyotypic studies, it was suggested that the karyotype origin of *T. breyeri* and *T. eratyrusiformis*, both with 2n = 24 chromosomes, was due to one X chromosome fission event in the ancestors of the *spinolai* group [36,38]. Our results support the origin of the 2n = 24 karyotype from the 2n = 23 ancestor and suggest that this modification in the number of chromosomes may have acted in the reproductive isolation between these lineages (Figure 1).

The next two possible cladogenetic events related to karyotypic changes occurred within the *infestans* clade (Figure 1). Our results suggest that the fission of one of the X chromosomes [event attributed to the karyotype origin of *T. vitticeps* (Stål, 1859) and *T. melanocephala* Neiva and Pinto, 1923 (both with 2n = 24 chromosomes) from the ancestral karyotype 2n = 23] [36,38] may have promoted reproductive isolation between the ancestors of the *vitticeps* group and those of other groups of the *infestans* clade (Figure 1). After that, strains with 23 chromosomes would continue to diverge and would undergo a new cladogenetic event, this time, by the fusion of the X_1_ and X_2_ chromosomes or the loss of one of the X sex chromosomes (becoming 2n = 20A + XY) (Figure 1). The lineage with 23 chromosomes would originate the species of the genus *Eratyrus* Stål, 1859, while the lineage with 22 chromosomes would originate the other species of the other groups. Experimental crosses have already been carried out between species of the *vitticeps* group and other groups from South America with 22 chromosomes (Table 1), and in all combinations, there was no hatching of eggs, confirming the role of the karyotype in the reproductive isolation and, consequently, in the diversification of some groups of South American triatomines.

In our analyses, only the chromosomal alteration that occurred in the ancestors of *T. maculata* (Erichson, 1848) was not related to a cladogenetic event (Figure 1). Based on our phylogeny and the phylogenetic reconstruction of Justi et al. [44], this species is positioned at the base of the *infestans* clade. Considering the ancestral karyotype 2n = 23 recovered in our analysis (PP > 0.75), we suggest that fusion (or loss) of an X chromosome may have occurred during the diversification of *T. maculata*. On the other hand, analyses with ultraconserved elements recovered this species between the *infestans* and the *brasiliensis* groups (both with 2n = 22 chromosomes) [48], so that the karyotype of *T. maculata* would not have changed in relation to the ancestor. Thus, further studies with this species may contribute to the understanding of these phylogenetic relationships.

### 2.4. Chromosomal Changes as a Reproductive Barrier

Some chromosomal changes have already been suggested as a mechanism of isolation between the species of Triatominae, such as, for example, the difference in heterochromatin pattern [58]. However, the hybridization capacity observed between species that have different heterochromatin patterns, such as those of the *infestans* group (*T. infestans*, *T. delpontei* and *T. platensis*), does not support this hypothesis [30]. Furthermore, it has recently been suggested that 45S rDNA translocations between sex chromosomes and autosomes may also contribute to the reproductive isolation of triatomines [37]. The authors suggested that, possibly, hybrids between species with different patterns of 45S rDNA present on autosomes/sex chromosomes may have lower fertility due to unbalanced gamete production [37].

The role of chromosomal changes in reproductive isolation and, consequently, in the speciation process has been extensively discussed over the years, with emphasis on chromosomal inversions [59,60,61,62,63,64,65]. Initially, it was believed that the crossing between polymorphic individuals for chromosomal changes would produce heterozygous hybrids, which would be sterile [65,66]. However, this has been questioned, since if these chromosomal changes promoted strong isolation, it would be difficult for this characteristic to be fixed in the population and, on the other hand, if the isolation was weak, it would be difficult for it to lead to speciation [65,66]. Subsequently, it was proposed that reproductive isolation would result from the suppression of recombination in areas where these inversions occurred, so that genes in these regions could differentiate, leading to the accumulation of divergence and, consequently, speciation [65]. Despite this, there is still no consensus on how and if, in fact, chromosomal speciation can occur.

On the other hand, most chromosomal speciation studies have been carried out with organisms that have monocentric chromosomes [61,65]. Fissions in this type of chromosome are more difficult to occur and to fixate in the population, due to the possibility that one of the fragments does not have centromeric regions and does not segregate correctly in meiosis [67]. Fusions can happen, but mainly between two acrocentric chromosomes (Robertsonian fusion) [67,68]. On the other hand, in holocentric chromosomes (which present the kinetochore diffuse along the chromosome), there is a greater facility for the occurrence of chromosomal fusion and fission [36,67].

In Triatominae, all changes related to a variation in the chromosome number involve fusion or fission events [36]. However, as mentioned several times in the manuscript, we do not have enough information to confirm whether fusion or just loss of one or more chromosomes has occurred. Recently, Pita et al. [52] suggested, based on the size of the sex chromosome X, that the karyotype 2n = 22 of *P. noireaui* is the result of a fusion event of the X chromosomes, from the ancestral karyotype 2n = 23. Furthermore, Alevi et al. [38] also suggested that the 2n = 21 karyotype of *P. megistus* is the result of a fusion event of a pair of autosomes, from the ancestral karyotype 2n = 23. We emphasize the importance of using appropriate techniques (such as chromosomal microdissection and the development of species-specific probes) to confirm whether the evolutionary event related to the chromosomal diversification of these *Panstrongylus* species was really associated with fusion or chromosomal loss.

Changes in chromosome number have already been related to reproductive isolation in organisms with holocentric chromosomes, such as plants [69] and, more recently, Lepidoptera [36]. The authors verified that for some groups of this order, these alterations may have played a role in cladogenesis among some species [36]. For triatomines, we could observe that nine cladogenetic events may be related to changes in the number of chromosomes (Figure 1, arrows). Adding these evolutionary events to the fact that in none of the experimental crosses between species with different karyotypes the eggs hatched (Table 1), we suggest that these alterations may play a role in the reproductive isolation of triatomines (we will call it karyotypic isolation) and can promote speciation (if fixed).

Considering the problems of chromosomal speciation (which also apply to karyotypic speciation), one of the suggested requirements for it to occur would be the population size [66], since in small populations, these changes would have more facility to become fixed by genetic drift [66]. The cladogenesis among some triatomine species (such as the *venosa* clade/remainder of Triatomini tribe, the *T. maculata*/*infestans* clade and *P. megistus*/*P. tibiamaculatus*) has been linked to vicariant events [44]. Thus, it is possible that in ancestral populations, with the emergence of a geographic barrier, this requirement was met, allowing the different karyotypes to be fixed and leading to cladogenesis (Figure 1).

## 3. Materials and Methods

### 3.1. Chromosomal Evolution Modeling

For the modeling study, a phylogenetic analysis with sequences of seven molecular markers obtained from GenBank for 88 triatomine species (Table S1) was initially performed (Figure S1). Molecular markers of four species of *Zelurus* spp. (Hemiptera, Reduviinae) were included as an outgroup, since this genus has been recovered close to Triatominae [44]. Although *Opisthacidius* spp. are closer to Triatominae [44], the species of this genus have never been studied cytogenetically. Thus, the choice of the taxa was mainly due to the availability of molecular markers and karyotype data for these species (Table S1). 

The sequences were aligned in the Mega11 program [70], using the Muscle method [71] and concatenated in Seaview4 [72]. The phylogenetic tree of Bayesian inference was reconstructed in the program BEAST 1.8.4 [73], under the substitution model GTR +I +G and Yule Process prior [74,75], in a total of 100 million generations. The burn-in was adjusted to 25% of the samples, and convergence (ESS > 200) was evaluated in Tracer 1.8 [76].

The resulting tree (Figure S1) was used as a basis for the analysis in the RevBayes v. 1.1.1. program [77], using the ChromoSSE model [42], to infer the ancestral karyotype at each node, as well as the karyotype changes that occurred anagenetically and cladogenetically along the phylogeny. The triatomine karyotypes used in the modeling study were obtained from Panzera et al. [36] and Reis and Alevi [39] (Table S1). In addition, the number of chromosomes considered for the *Zelurus* clade (2n = 22) was proposed based on the karyotypes of *Z. ochripennis* (Stål, 1854) and *Z. femoralis longispinis* Lent and Wygodzinsky, 1954 [78,79] (for which molecular data are not available), because unfortunately there are no cytogenetic data for the *Zelurus* species used in the phylogenetic analysis. The analyses were carried out with a total of 20,000 generations, with burn-in adjusted in 25% of the samples, and convergence (ESS > 200) was evaluated in Tracer 1.8 [76]. The resulting tree was then plotted in R 4.2 [80], using the ggtree [81,82,83,84,85] and Revgadgets [86] packages. Vertical bars and grouping names were inserted using Adobe Illustrator CS6.

### 3.2. Experimental Crosses

To assess the reproductive compatibility between species with different chromosome numbers, three crosses were performed for each couple, as shown in Table 1. For each species used, intraspecific crosses were also performed (control). However, due to the low availability of live insects, intraspecific crosses between *P. lecticularia*, *T. longipennis* and *T. rubrovaria* (Blanchard, 1843) were not performed.

The species used were provided by the Triatominae Insectarium of the School of Pharmaceutical Sciences (FCFAR/UNESP), Araraquara, São Paulo, Brazil, where the crossings were also carried out. We emphasize that the choice of the species was based on the availability of live insects with different karyotypes kept in the FCFAR/UNESP insectarium.

To ensure the virginity of the tested insects, fifth instar nymphs were separated and sexed. After reaching the adult stage, the crosses were initiated and lasted 4 months. Insect feeding and oviposition counting were performed weekly during this period. The insects were kept at room temperature (average of 24 °C) and relative humidity of 63% [87]. After the crossing period, the eggs were kept for another two months to check the hatching rate.

## 4. Conclusions

Based on the above, we can conclude that: i. the ancestral karyotype of Triatominae is 2n = 22 chromosomes; ii. during the evolutionary process, at least nine cladogenetic events associated with alterations in the number of chromosomes may have occurred in triatomines; iii. these alterations could act as a prezygotic barrier in Triatominae (karyotypic isolation) and, consequently, promote species diversification; and iv. the description of new karyotypes (for example, species of the genus *Linshcosteus*, the Old World *Triatoma*, species of the Alberprosini, Cavernicolini and Bolboderini tribes and reduvids, phylogenetically close to Triatominae), the use of new molecular markers, the development of species-specific probes by chromosomal microdissection, and carrying out studies of experimental crosses can contribute to the elucidation of the evolutionary history of this group of vectors.

Finally, we emphasize that some phylogenetic relationships need to be better elucidated, namely, i. the position of *T. maculata* in relation to the *brasiliensis* group; ii. the position of *T. guasayana* in relation to the *sordida* group; iii. the position of the *spinolai* group in relation to the North and South American lineages; iv. the position of the *Eratyrus* group in relation to the *infestans* clade; and v. the position of the *rufotuberculatus* group in relation to the *geniculatus* clade, since the species of this group were recovered closer to the *rubrofasciata* clade (genus *Triatoma*).

## Figures and Tables

**Table 1:** Number of eggs and hatching rate resulting from experimental crosses between species with different chromosome numbers. CN: diploid chromosome number.

Experimental Crosses			Number of Eggs	Hatching Rate (%)	Reference
Female (CN)	x	Male (CN)			
Interspecific cross					
*T. longipennis* (23)	x	*T. vitticeps* (24)	26	0	This paper
*T. vitticeps* (24)	x	*T. longipennis* (23)	72	0	This paper
*T. longipennis* (23)	x	*T. infestans* (22)	38	0	This paper
*T. infestans* (22)	x	*T. longipennis* (23)	45	0	This paper
*T. infestans* (22)	x	*T. protracta* (23)	146	0	This paper
*T. protracta* (23)	x	*T. infestans* (22)	93	0	This paper
*T. protracta* (23)	x	*P. lecticularia* (22)	160	0	This paper
*P. lecticularia* (22)	x	*T. protracta* (23)	82	0	This paper
*P. tibiamaculatus* (23)	x	*T. brasiliensis* (22)	60	0	This paper
*T. brasiliensis* (22)	x	*P. tibiamaculatus* (23)	193	0	This paper
*T. pseudomaculata* (22)	x	*P. tibiamaculatus* (23)	150	0	This paper
*P. tibiamaculatus* (23)	x	*T. pseudomaculata* (22)	102	0	This paper
*T. melanocephala* (24)	x	*P. tibiamaculatus* (23)	102	0	This paper
*P. tibiamaculatus* (23)	x	*T. melanocephala* (24)	237	0	This paper
*T. rubrovaria* (22)	x	*P. tibiamaculatus* (23)	21	0	This paper
*P. tibiamaculatus* (23)	x	*T. rubrovaria* (22)	53	0	This paper
*T. infestans* (22)	x	*P. tibiamaculatus* (23)	174	0	This paper
*P. tibiamaculatus* (23)	x	*T. infestans* (22)	93	0	This paper
*T. brasiliensis* (22)	x	*T. vitticeps* (24)	90	0	[18]
*T. vitticeps* (24)	x	*T. brasiliensis* (22)	147	0	[18]
*T. melanocephala* (24)	x	*T. brasiliensis* (22)	78	0	[18]
*T. brasiliensis* (22)	x	*T. melanocephala* (24)	63	0	[18]
*P. megistus* (21)	x	*P. tibiamaculatus* (23)	107	0	[32]
*P. tibiamaculatus* (23)	x	*P. megistus* (21)	265	0	[32]
*P. megistus* (21)	x	*P. lignarius* (23)	157	0	[32]
*P. lignarius* (23)	x	*P. megistus* (21)	523	0	[32]
Intraspecific cross					
*T. infestans*	x	*T. infestans*	439	62	This paper
*T. protracta*	x	*T. protracta*	278	86	This paper
*P. lignarius*	x	*P. lignarius*	700	51	[32]
*P. megistus*	x	*P. megistus*	372	68	[32]
*P. tibiamaculatus*	x	*P. tibiamaculatus*	190	65	[18]
*T. brasiliensis*	x	*T. brasiliensis*	271	59	[18]
*T. melanocephala*	x	*T. melanocephala*	302	63	[18]
*T. vitticeps*	x	*T. vitticeps*	353	70	[18]

## Data Availability

All relevant data are within the manuscript.

## Supplementary Materials

The following supporting information can be downloaded at https://www.mdpi.com/article/10.3390/ijms24076350/s1.
