# Supplementary figures and images for: Karyotype Evolution in Triatominae (Hemiptera, Reduviidae): The Role of Chromosomal Rearrangements in the Diversification of Chagas Disease Vectors
Source: Int J Mol Sci. 2023 Mar 28;24(7):6350. doi: 10.3390/ijms24076350 (PMC10094360; doi:10.3390/ijms24076350)

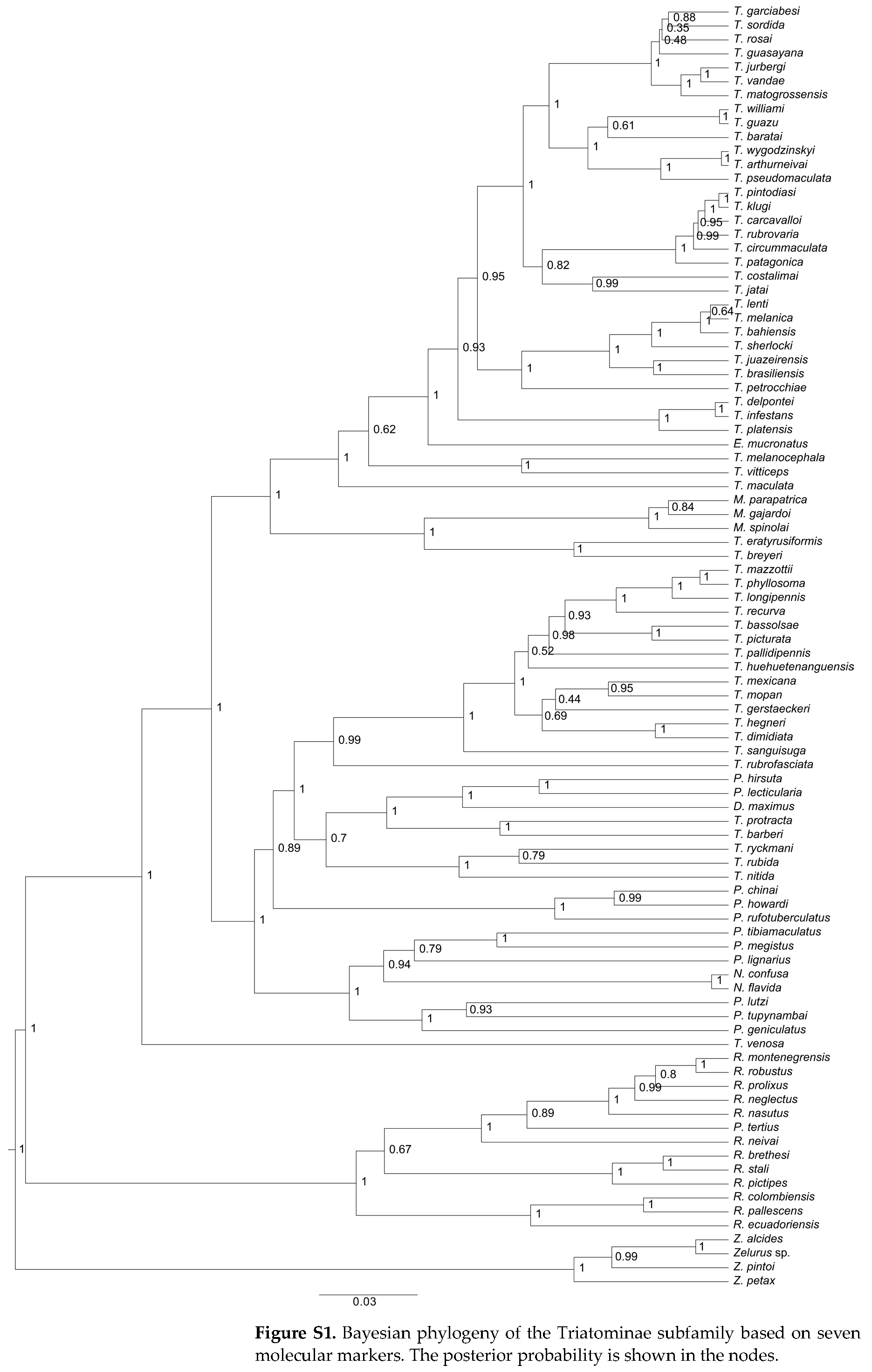

Supplement: Supplementary file 1 [file ijms-24-06350-s001.zip › Figure S1.png]
