# Supplementary material for: Karyotype Evolution in Triatominae (Hemiptera, Reduviidae): The Role of Chromosomal Rearrangements in the Diversification of Chagas Disease Vectors
Source: Int J Mol Sci. 2023 Mar 28;24(7):6350. doi: 10.3390/ijms24076350 (PMC10094360; doi:10.3390/ijms24076350)
Supplement: Supplementary file 1 [file ijms-24-06350-s001.zip › Table S1.doc]

**Table S1.** GenBank accession code and karyotype for each species. Number of chromosomes obtained from Panzera et al. [36] and Reis; Alevi [39].

| **Species** | **Molecular markers** | | | | | | | **Chromosome number** |
| --- | --- | --- | --- | --- | --- | --- | --- | --- |
|  | ***Cytb*** | ***16S*** | ***18S*** | ***28S*** | ***COI*** | ***COII*** | ***ITS2*** |  |
| *D. maximus* | KC249226 | KC248968 | KC249092 | KC249134 | KC249306 | KC249399 | AJ286887 | 22 |
| *E. mucronatus* |  | JQ897794 | JQ897555 | JQ897635 | AF449140 |  | EF550126 | 23 |
| *M. gajardoi* | JN102359 |  |  |  | GQ336896 |  |  | 23 |
| *M. parapatrica* | MN117884 |  |  |  | MN117870 |  |  | 23 |
| *M. spinolai* | JN102358 | AF324518 | AJ421961 |  | MN117874 |  |  | 23 |
| *N. confusa* |  | KC248989 |  | KC249146 |  | KC249418 |  | 23 |
| *N. flavida* | JX848648 | AY035451 | AJ421959 |  |  |  | AM286732 | 23 |
| *P. chinai* | JX400960 |  |  |  | MN504941 | MN504970 | AJ306547 | 23 |
| *P. geniculatus* | KX109903 | AF394593 | JQ897583 | KX109907 | MW983060 |  |  | 23 |
| *P. hirsuta* |  | FJ230443 | FJ230521 | FJ230604 |  |  |  | 22 |
| *P. howardi* | JX400969 |  |  |  |  | MN504980 | MN505070 | 23 |
| *P. lecticularia* | MT702379 | KC249029 | KC249111 | KC249176 | NC_050326 | NC_050326 | AY860407 | 22 |
| *P. lignarius* | MK829849 | AY185833 | JQ897584 | KX109906 | AF449141 |  | AJ306549 | 23 |
| *P. lutzi* | KC249227 | AY035449 |  | KC249135 | KC249307 | KC249401 |  | 24 |
| *P. megistus* | KC249228 | KC248970 | AJ243336 | KC249137 | KC249308 | KC249402 | AJ306542 | 21 |
| *P. rufotuberculatus* | JX400989 | KY748239 | AJ421955 |  | MF614953 | MF614953 | AJ306546 | 23 |
| *P. tertius* |  | AY035439 | Y18751 |  |  |  | AJ286891 | 22 |
| *P. tibiamaculatus* | KC249296 | KC249081 | KC249127 | KC249214 | KC249389 | KC249485 |  | 23 |
| *P. tupynambai* | KC249233 | KC248978 |  | KC249142 | MZ643677 | KC249404 |  | 23 |
| *R. brethesi* | KC249235 | KC248980 |  |  | KC249313 | KC249405 |  | 22 |
| *R. colombiensis* | FJ229360 | AY035438 |  | KC543516 |  |  | KT351069 | 22 |
| *R. ecuadoriensis* | AF045715 | AF028746 |  | KC543518 |  | GQ869665 | KT267950 | 22 |
| *R. montenegrensis* | KR072682 |  |  |  |  |  |  | 22 |
| *R. nasutus* | JX273155 | AF028749 |  | AF435856 |  |  | KT317022 | 22 |
| *R. neglectus* | KT317068 | JQ897839 | JQ897601 | JQ897670 |  |  | KT317033 | 22 |
| *R. neivai* |  | AY035441 |  |  | AF449137 |  |  | 22 |
| *R. pallescens* | EF071584 |  |  | KC543527 |  |  | KT351070 | 22 |
| *R. pictipes* | KC249236 | JQ897840 | KC249093 |  | KC249314 | KC249407 | FJ887796 | 22 |
| *R. prolixus* | EF043579 | AF028747 | AJ421962 | AF435862 | AF449138 | NC_050328 | AJ286888 | 22 |
| *R. robustus* | AF421343 | MF966360 |  | MF966331 |  |  | MK411278 | 22 |
| *R. stali* | KC249237 | KC248984 | KC249095 | KY111699 | KC249317 | KC249410 | FJ887794 | 22 |
| *T. arthurneivai* |  | AY035460 |  |  |  |  | AM286736 | 22 |
| *T. bahiensis* | KT347298 |  |  |  |  |  |  | 22 |
| *T. baratai* | KC249238 | KC571991 |  | KC249143 |  | KC249411 |  | 22 |
| *T. barberi* | MT556655 | JX872242 | AJ421958 |  | MT556655 | MT556655 | AJ293590 | 23 |
| *T. bassolsae* | AY859410 |  |  |  |  |  | MK248256 | 23 |
| *T. brasiliensis* | KC249239 | KC248985 | AJ421957 | KC249145 | KC249318 | KC249413 | AJ293591 | 22 |
| *T. breyeri* | KC249242 | KC248988 |  |  | KC249321 | KC249417 |  | 24 |
| *T. carcavalloi* | KC249244 | KC248990 | KC249097 |  | KC249322 | KC249419 |  | 22 |
| *T. circummaculata* | KC249245 | KC248992 | KC249099 | KC249148 | KC249325 | KC249422 |  | 22 |
| *T. costalimai* | KC249246 | KC248997 | KC249101 | KC249149 | KC249327 | KC249425 |  | 22 |
| *T. delpontei* | KC249248 | KC249000 |  | KC249150 | KC249330 | KC249427 | AJ576060 | 22 |
| *T. dimidiata* | FJ197155 | KC249004 | JQ897609 | KC249152 |  | KC249431 | AM286693 | 23 |
| *T. eratyrusiformis* | JN102360 | AY035466 |  |  | GQ336898 |  | FN396537 | 24 |
| *T. garciabesi* | KC249249 | KC249006 | KC249102 | KC249158 | KC249338 | KC249434 |  | 22 |
| *T. gerstaeckeri* | JQ282723 |  |  | KF188642 | MT587783 |  | AM286734 | 23 |
| *T. guasayana* | KC249252 | KC249012 | KC249103 | KC249162 | KC249343 | KC249438 |  | 22 |
| *T. guazu* | KC608976 | KC249013 | KC249105 | KC249164 | KC608984 | KC249440 |  | 22 |
| *T. hegneri* | JN585830 |  |  |  |  |  | AM286727 | 23 |
| *T. huehuetenanguensis* | MG951755 |  |  |  | NC_050325 | NC_050325 | MG947606 | 23 |
| *T. infestans* | KC249256 | KC249016 | KC249109 | KC249168 | KC249349 | KC249442 | AJ289876 | 22 |
| *T. jatai* |  | KT601153 |  |  | KT601164 |  |  | 22 |
| *T. juazeirensis* | KC249263 | KC249026 |  | KC249173 | KF826892 |  |  | 22 |
| *T. jurbergi* | KC249264 | KC249027 | KC249110 | KC249174 |  | KC249448 |  | 22 |
| *T. klugi* | KC249265 | KC249028 |  |  | KC249356 | KC249449 |  | 22 |
| *T. lenti* | KY576789 | KY576788 |  |  | KY576792 |  |  | 22 |
| *T. longipennis* | KC249267 | KC249031 | AJ243331 | KC249177 | KC249357 | MT556658 | AJ286883 | 23 |
| *T. maculata* | KC249268 | KC249035 |  | KC249178 | AF449139 | KC249455 | AJ582027 | 22 |
| *T. matogrossensis* | KC249269 | KC249036 | KC249114 | KC249179 | KC249359 | KC249456 |  | 22 |
| *T. mazzottii* | DQ198816 | AY035446 | AJ243333 |  | NC_050327 | NC_050327 | AY860393 | 23 |
| *T. melanica* |  | KC249041 |  | KC249183 | KJ580495 | KC249461 |  | 22 |
| *T. melanocephala* | KF826898 | KF769450 |  |  | KF826895 |  |  | 24 |
| *T. mexicana* | DQ118976 | JX872251 |  |  | NC_050324 | NC_050324 | AM286728 | 23 |
| *T. mopan* | JN585883 |  |  |  |  |  | MG954252 | 23 |
| *T. nitida* | AF045723 | JX872239 |  |  | MT556667 | MT556667 | JX872260 | 21 |
| *T. pallidipennis* | EU790632 | KC249044 | KC249115 | KC249184 | MT556659 | MT556659 | AM286730 | 23 |
| *T. patagonica* | MG241451 | AY035464 |  |  | KR139999 |  |  | 22 |
| *T. petrocchiae* | KY654075 | KY654073 |  |  | KY654074 |  |  | 22 |
| *T. phyllosoma* | DQ198818 |  | AJ243329 |  | MT556660 | MT556660 | AJ286881 | 23 |
| *T. picturata* | DQ198817 | AY185840 | AJ243332 |  | MT556661 | MT556661 | AJ286884 | 23 |
| *T. pintodiasi* |  | MG264738 |  |  | MZ345607 |  |  | 22 |
| *T. platensis* | KC249274 | KC249047 |  | KC249186 | KC249363 | KC249462 | AJ576062 | 22 |
| *T. protracta* | MT239325 | KC249048 | KT231850 | KC249187 | MT556662 | MT556662 | JX872263 | 23 |
| *T. pseudomaculata* | KC249275 | KC249051 |  | KC249189 | KC249366 | KC249464 |  | 22 |
| *T. recurva* | DQ198813 | FJ230417 | FJ230496 | FJ230577 | MT556663 | MT556663 | MK248251 | 23 |
| *T. rosai* | KC249295 | KC249078 |  | KC249213 | MH029697 |  |  | 22 |
| *T. rubida* | DQ198809 | AY035445 |  | GQ853391 | MT556664 | MT556664 | AM286735 | 23 |
| *T. rubrofasciata* | MH368021 | AY127046 | AJ421960 | KR632546 | MH934953 | MH934953 |  | 25 |
| *T. rubrovaria* | KC249281 | KC249067 | KC249117 | KC249198 | KC249370 | KC249471 | AJ557261 | 22 |
| *T. ryckmani* |  | JX872248 |  |  |  |  | AM286731 | 23 |
| *T. sanguisuga* | HQ141317 | JX890269 |  | GQ853392 | NC_050329 | NC_050329 | KF142511 | 23 |
| *T. sherlocki* | KC249288 | KC249068 |  | KC249205 | KC249377 | KC249478 |  | 22 |
| *T. sordida* | MH054940 |  |  | KC249210 | MH029692 |  |  | 22 |
| *T. vandae* | KC249298 | KC249083 | KC249128 | KC249216 | KC249391 | KC249487 |  | 22 |
| *T. venosa* |  | JQ897850 | JQ897611 | JQ897681 |  |  | AJ582026 | 22 |
| *T. vitticeps* | KC249303 | KC249087 | KC249132 | KC249220 | KC249396 |  |  | 24 |
| *T. williami* | KC608981 | KC249089 |  |  | KC608990 | KC249493 |  | 22 |
| *T. wygodzinskyi* |  | KC249090 | KC249133 | KC249222 | KC249398 | KC249494 |  | 22 |
| *Z. alcides* |  | JQ897855 | JQ897615 | JQ897686 |  |  |  | 22* |
| *Z. petax* |  | FJ230416 | FJ230495 | FJ230576 | JQ888708 |  |  | 22* |
| *Z. pintoi* |  | JQ897856 | JQ897616 | JQ897687 |  |  |  | 22* |
| *Zelurus* sp*.* | GQ869679 | JQ897857 | JQ897618 |  | JQ888708 | GQ869673 |  | 22* |

*Inferred based on Poggio et al. [78] e Oliveira et al. [79].
